# Deceleration capacity derived from a five-minute electrocardiogram predicts mortality in the general population

**DOI:** 10.1038/s41598-024-83712-w

**Published:** 2024-12-19

**Authors:** Alexander Steger, Petra Barthel, Alexander Müller, Ina-Maria Rückert-Eheberg, Birgit Linkohr, Julia Allescher, Melanie Maier, Alexander Hapfelmeier, Eimo Martens, Helene Hildegard Heidegger, Arne Michael Müller, Konstantinos D. Rizas, Stefan Kääb, Moritz F. Sinner, Daniel Sinnecker, Karl-Ludwig Laugwitz, Annette Peters, Georg Schmidt

**Affiliations:** 1 Department of Internal Medicine I, TUM University Hospital, Technical University of Munich, Ismaninger Str. 22, 81675 Munich, Germany; 2 German Centre for Cardiovascular Research (DZHK), Partner Site Munich Heart Alliance, Munich, Germany; 3 Institute of Epidemiology, Helmholtz Zentrum München, German Research Center for Environmental Health, Munich-Neuherberg, Germany; 4 Institute of AI and Informatics in Medicine, TUM School of Medicine and Health, Technical University of Munich, Munich, Germany; 5 Institute of General Practice and Health Services Research, TUM School of Medicine and Health, Technical University of Munich, Munich, Germany; 6 Department of Obstetrics and Gynecology, LMU University Hospital, LMU Munich, Munich, Germany; 7 Department of Cardiology, LMU University Hospital, LMU Munich, Munich, Germany; 8 MVZ Harz, Goslar, Germany; 9 Institute for Medical Information Processing, Biometry and Epidemiology, LMU Munich, Munich, Germany

**Keywords:** General population screening, Non-invasive long-term risk stratification, Fully automated biosignal analysis, Electrocardiogram, Deceleration capacity of the heart rate, Autonomic regulation, Population screening, Predictive markers, Prognosis, Cardiology, Computational biology and bioinformatics, Outcomes research, Scientific data

## Abstract

In contemporary healthcare, effective risk stratification in the general population is vital amidst rising chronic disease rates and an ageing demographic. Deceleration Capacity of the heart rate (DC), derived from 24-hour Holter electrocardiograms, holds promise in risk stratification for cardiac patients. However, the potential of short-term electrocardiograms of five minutes duration for population screening has not been fully explored. Our study aims to investigate the utility of Deceleration Capacity derived from short-term electrocardiograms as a scalable, fully-automated screening tool for predicting long-term mortality risk in the general population. Within a cohort of a representative population-based survey in Germany (KORA-KMC-study), 823 participants with sinus rhythm aged 27 to 76 years at enrollment (females 47.4%) were followed for a median of 13.4 years (IQR 13.1–13.6). All-cause mortality was defined as the primary endpoint and observed in 159 participants. Deceleration Capacity was calculated from 5-minute 12-lead electrocardiograms by a fully automated approach. Participants were divided into three predefined risk categories: DC_category0_ – low-risk (> 4.5ms); DC_category1_ – intermediate-risk (2.5-4.5ms); and DC_category2_ – high-risk (≤ 2.5ms). More than two-thirds of the participants (*n* = 564, 68.5%) fell into DC_category0_, about one-fifth (*n* = 168, 20.4%) into DC_category1_, and about one-tenth (*n* = 91, 11.1%) into DC_category2_. Estimated 13-years mortality in the risk groups was 16.7%, 23.5%, and 49.1%, respectively (*p* < 0.001). Adjusting for age, life-style-related risk factors, and comorbidities, increased mortality risk was observed for DC_category2_ (HR 2.34, 95%-CI 1.56–3.50). Deceleration Capacity, derived automatically from brief 5-minute electrocardiogram recordings, emerges as a robust, feasible, and independent predictor of long-term mortality risk in the general population.

**Supplementary Information:**

The online version contains supplementary material available at 10.1038/s41598-024-83712-w.

## Introduction

In an ageing population with an increasing burden of chronic disease, risk stratification in the general population has become central to modern medicine. Through the use of non-invasive screening methods such as imaging techniques, biomarkers, and risk prediction models, healthcare professionals aim to better assess individual health risk and tailor interventions accordingly to improve overall health outcomes and to reduce healthcare costs.

The Deceleration Capacity of the heart rate (DC) is a non-invasive risk predictor that has gained considerable attention in the field of cardiovascular research. It represents the ability of the heart rate to decelerate, reflecting the autonomic regulation of the cardiovascular system^1^. Deceleration Capacity has been shown to provide valuable insight into the overall health and autonomic function of the heart, making it a promising tool for risk stratification and prognostic assessment in patients suffering from various cardiovascular conditions^2–7^. In the general population, the long-term prognostic power of Deceleration Capacity as a potential screening parameter has not been investigated so far. Initially, Deceleration Capacity has been derived from 24 h Holter electrocardiograms limiting its scalability as a simple screening method for the general population. However, convincing evidence exists, that the duration of the electrocardiogram recording can be significantly shorter without losing prognostic power^1,2^. Furthermore, Deceleration Capacity assessment originally included manual electrocardiogram editing by experienced professionals to obtain an accurate sequence of RR intervals limiting again the scalability in a comprehensive screening context.

The aim of this study was to derive Deceleration Capacity from short-term electrocardiogram recordings of five minutes’ duration using a fully automated approach and to evaluate it as an independent predictor of long-term mortality risk in the general population.

## Methods

### Population and follow-up

The KORA study (Cooperative Health Research in the Region of Augsburg) is a representative large-scale population-based research project conducted in the city of Augsburg and in two surrounding rural districts in southern Germany. Participants were of German nationality and were randomly selected from the population registers of the study region, stratified by sex and age. KORA aims to investigate the causes and consequences of common chronic diseases such as diabetes, cardiovascular and respiratory diseases. The study collects comprehensive data on various health-related factors, including clinical examination results, cardiovascular risk factors, medical history and medications. The assessment of alcohol intake (grams per day) is based on self-reported recall of weekday and weekend consumption of beer, wine and spirits^8^. A specific cohort within the KORA study is the KORA/MAGiC Control Cohort (KMC), which was established in 2002^9^. In this cohort, participants were enrolled and followed up with regular standardized study visits, with the last morbidity and mortality follow-up in 2016. In case a participant could not be traced, the population registry was used to identify those who died. The study was conducted in accordance with the tenets of the Declaration of Helsinki. Written informed consent was obtained from all participants and the study was approved by the Ethics Committee of the Bavarian Medical Association (KMC EC No. 01216 and GEFU4 EC No. 08064).

### Deceleration Capacity assessment

A 5-minute 12-lead resting electrocardiogram (Portrait, Mortara, Milwaukee, Wisconsin, USA) was recorded in all participants. Participants with atrial fibrillation were excluded from the study.

The electrocardiograms were digitally stored and analyzed using a fully automated approach, eliminating the need for manual editing. Filtering techniques were applied to all leads to remove segments with insufficient signal quality (noise), including those lacking a clear signal (loss of contact) or exceeding the amplitude resolution. For noise detection, the measurements were divided into segments of 5 s. The electrocardiogram signal was anticipated within a frequency range of 2 to 45 Hz. Segments with a large high-frequency component were classified as noise. This was defined by calculating the total summed power of the power spectrum in the range of 2 –45 Hz (“A”) and the total summed power in the range > 45 Hz (“B”). A noise-to-signal ratio (Q = B/A) was then calculated. Q > 0.4 was considered as insufficient signal quality due to noise. Loss of contact segments were defined when the standard deviation of the signal amplitudes was < 50 µV. The corresponding segments were excluded from the further analyses.

An electrocardiogram lead was only utilized for further processing if the proportion of noise was less than 10% of the total measurement. If noise levels exceeded 10% in all leads, preference was given to the lead exhibiting the lowest noise level. The open-source software libRASCH was applied to the selected electrocardiogram leads to annotate the QRS complexes and derive the correct sequence of RR intervals from the raw electrocardiogram signals^10^. Only RR interval durations ranging from 300ms to 2,500ms were considered for assessing Deceleration Capacity.

Deceleration Capacity characterizes the average capacity of the heart to decelerate the cardiac rhythm from one beat to the next. The technical details of Deceleration Capacity assessment (MATLAB, Mathworks, Natick, Massachusetts, USA) by using the phase-rectified signal averaging method (PRSA) have been described elsewhere^1,11,12^. Briefly, beat to beat RR interval prolongations (heart rate decelerations) were identified as so-called anchors. Segments of interval data around the anchors were selected and aligned (phase rectified) at the anchors. The PRSA signal is obtained by averaging the RR interval data within the aligned segments. The central part of the PRSA signal is then quantified by Haar wavelet analysis to obtain an estimate of Deceleration Capacity. If multiple leads were utilized to calculate Deceleration Capacity, the median derived from all utilized leads was used. In accordance with previous studies, patients were stratified to three distinct risk categories: DC_category 0_ - low risk (> 4.5 ms); DC_category 1_ - intermediate risk (2.5–4.5 ms); and DC_category 2_ - high risk (≤ 2.5 ms)^1,2^.

### Statistics

Continuous variables are presented as medians with interquartile ranges, while categorical variables are presented as absolute and relative frequencies. The Mann-Whitney U test was used for hypothesis testing for group differences in continuous variables. The mortality risk 13 years after enrollment was estimated using the Kaplan-Meier method. The time-dependent receiver operating characteristics (ROC) curve for the prediction of all-cause mortality within 13 years by Deceleration Capacity was calculated, plotting the relation of dynamic specificity to cumulative sensitivity in dependence of Deceleration Capacity^13^. The area under the curve (AUC) was used as performance measure, i.e., measure of discrimination. Cox proportional hazards models were used, and hazard ratios with 95% confidence intervals were reported. Hypothesis testing was performed using z-tests for association between variables and right-censored outcome measures. Two multivariable models were employed to assess the prognostic significance of Deceleration Capacity while accounting for potential confounders and for modifying effects of pre-existing cardiometabolic conditions. Multivariable model 1 included age, median heart rate, and conventional external risk factors (i.e., smoking status, alcohol consumption, and body mass index). In multivariable model 2, pre-existing cardiometabolic conditions (specifically, diabetes mellitus and a history of myocardial infarction) were used as additional covariates. Further multivariable models were applied to investigate the interaction between Deceleration Capacity and cardiometabolic conditions, age, heart rate, alcohol consumption, and smoking status. Datasets were complete for 792 study participants. From the remaining 31 individuals, 4 had missing data on alcohol consumption, 9 had missing data on body mass index, and 19 had missing data on smoking habits (one participant did not provide information on both smoking habits and alcohol consumption). To ensure comprehensive data analysis, multiple imputation by polyconditional specification was applied. The imputation included the following variables: age, sex, survival information, alcohol consumption, smoking habits, body mass index, diabetes mellitus, previous myocardial infarction, median heart rate, and Deceleration Capacity. Cox proportional hazards models were performed with continuous values of Deceleration Capacity as well as with categorical values of Deceleration Capacity (categories 0, 1, 2). Using three Deceleration Capacity risk categories, Kaplan-Meier survival curves were calculated for the low-risk, the intermediate-risk, and the high-risk subgroups. The log-rank test was used to compare Kaplan-Meier survival curves. All hypothesis tests were two-sided and performed at a 5% significance level. Analyses were performed using IBM SPSS Statistics, version 28 (IBM Corp, Armonk, NY, USA) and R 4.3.1 (The R Foundation for Statistical Computing, Vienna, Austria).

## Results

A total of 833 participants were enrolled in this cohort, of whom 3 were lost to follow-up. 7 participants were excluded due to overt atrial fibrillation. The resulting 823 participants, aged 27 to 76 years at enrollment, were followed for a median of 13.4 years (IQR 13.1–13.6). Table 1 shows selected baseline clinical characteristics of the included participants. The sex distribution was balanced with 47.4% female participants. The prevalence of cardiovascular risk factors in the studied cohort was as follows: 66 participants (8.0%) with diabetes mellitus, 362 participants (44.0%) with arterial hypertension, 416 participants (50.6%) with hyperlipidemia, 491 (59.7%) current or former smokers. The median value of Deceleration Capacity in the KORA KMC cohort was 6.1 ms (IQR 3.9–8.5 ms).

**Table 1:** Clinical characteristics of the study population.

Variables	KORA KMC cohort
Number of participants, n (%)	823 (100)
Age (years), median (IQR)	63 (57–70)
Female, n (%)	390 (47.4)
BMI (kg/m^2^), median (IQR)	27 (25–30)
History of AMI, n (%)	40 (4.9)
History of PAD, n (%)	95 (11.5)
History of Stroke, n (%)	25 (3.0)
Diabetes mellitus, n (%)	66 (8.0)
Hypertension, n (%)	362 (44.0)
Active or former smoker, n (%)	491 (59.7)
Hyperlipidemia, n (%)	416 (50.6)
COPD, n (%)	111 (13.5)
Asthma, n (%)	74 (9.0)
ACE-Inhibitors, n (%)	114 (13.9)
AT1-receptor antagonist, n (%)	50 (6.1)
Calcium antagonists, n (%)	70 (8.5)
Betablockers, n (%)	166 (20.2)
Diuretics, n (%)	147 (17.9)
Statins, n (%)	104 (12.6)
Heart rate (min^− 1^), median (IQR)	66 (59–73)
DC (ms), median (IQR)	6.1 (3.9–8.5)

IQR, interquartile range; BMI, body mass index; AMI, acute myocardial infarction; PAD, peripheral artery disease; COPD, chronic obstructive pulmonary disease; ACE, angiotensin-converting enzyme; DC, Deceleration Capacity.

**Fig. 1:**
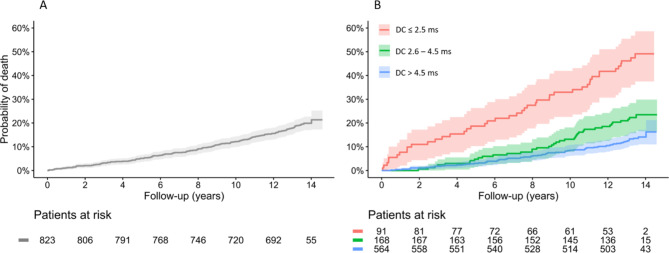
Kaplan-Meier estimates of mortality (solid lines) with 95% confidence intervals (shaded areas) of the complete KORA KMC cohort (**A**) and stratified by three Deceleration Capacity risk groups (**B**). (**B**) Probabilities of mortality were significantly different between all three Deceleration Capacity risk groups (*p* < 0.001). DC, Deceleration Capacity.

During the follow-up period, 159 events were observed. The estimated mortality risk 13 years after enrollment was 21.3%. The Kaplan-Meier curve of the study population is shown in Fig. 1A.

Older age (dichotomized at 65 years, HR 5.84, 95%-CI 3.92–8.68), presence of diabetes mellitus (HR 2.35, 95%-CI 1.54–3.61), a history of myocardial infarction (HR 2.98, 95%-CI 1.83–4.88), an elevated baseline median heart rate (dichotomized at 75 min^− 1^, HR 1.52, 95%-CI 1.07–2.16), and alcohol consumption habits (dichotomized at an intake of 20 g/d, HR 1.16, 95%-CI 1.04–1.28) were significantly associated with an increased mortality risk. However, smoking status and body mass index were not significantly associated with mortality in the KORA KMC cohort (Table 2, univariable model). In those participants who died during follow-up, the median value of Deceleration Capacity was 4.4 ms (IQR 2.1–6.6 ms) compared to 6.5 ms (IQR 4.4–9.0) in the remaining participants (*p* < 0.001).

**Table 2:** Univariable and multivariable Cox proportional hazards models for the prediction of mortality.

Variable	Univariable models		Multivariable model 1		Multivariable model 2	
	HR (95%-CI)	*p*	HR (95%-CI)	*p*	HR (95%-CI)	*p*
Age ≥ 65 years	5.84 (3.92–8.68)	< 0.001	6.54 (4.25–10.1)	< 0.001	6.17 (4.00–9.51)	< 0.001
Diabetes mellitus	2.35 (1.54–3.61)	< 0.001	Not part of the model		1.19 (0.75–1.90)	0.453
Previous AMI	2.98 (1.83–4.88)	< 0.001	Not part of the model		2.20 (1.32–3.65)	0.002
Mean heart rate ≥ 75 min^− 1^	1.52 (1.07–2.16)	0.021	1.43 (0.98–2.10)	0.063	1.43 (0.98–2.08)	0.066
Body mass index, kg/m^2^	1.03 (0.99–1.07)	0.077	1.01 (0.97–1.05)	0.705	1.00 (0.96–1.04)	0.935
Active smoker	1.56 (1.00–2.44)	0.049	2.32 (1.47–3.69)	< 0.001	2.29 (1.44–3.63)	< 0.001
Former smoker	1.36 (0.96–1.93)	0.087	1.30 (0.90–1.86)	0.162	1.23 (0.86–1.77)	0.265
Alcohol consumption, 20 g/day	1.17 (1.04–1.32)	0.012	1.22 (1.00–1.49)	0.001	1.22 (1.00–1.49)	0.001
DC_category 1_	1.74 (1.18–2.60)	0.005	1.10 (0.74–1.64)	0.635	1.14 (0.76–1.70)	0.527
DC_category 2_	4.61 (3.18–6.70)	< 0.001	2.34 (1.56–3.51)	< 0.001	2.34 (1.56–3.50)	< 0.001

HR, hazard ratio; 95%-CI, 95% confidence interval; AMI, acute myocardial infarction; min^–1^, beats per minute; DC, Deceleration Capacity; DC reference: DC _category 0_.

Based on the predefined Deceleration Capacity cut-off values, the study population was divided into three risk categories^1,2^. Deceleration Capacity was > 4.5 ms (low-risk, DC_category 0_) in 564 participants (68.5%), 2.6–4.5 ms (intermediate-risk, DC_category 1_) in 168 individuals (20.4%), and ≤ 2.5 ms (high-risk, DC_category 2_) in the remaining 91 individuals (11.1%). 77 deaths were observed in individuals with low-risk Deceleration Capacity (16.7%), 38 deaths in individuals with intermediate-risk Deceleration Capacity (23.5%), and 44 deaths in individuals with high-risk Deceleration Capacity (49.1%). The corresponding Kaplan-Meier curves are shown in Fig. 1B. The probabilities of mortality were significantly different between all three Deceleration Capacity risk groups (*p* < 0.001). The long-term mortality risk prediction by Deceleration Capacity over a period of more than 10 years was substantiated using a landmark Kaplan-Meier analysis with a landmark time of 10 years (Supplementary Figure S1).

**Fig. 2:**
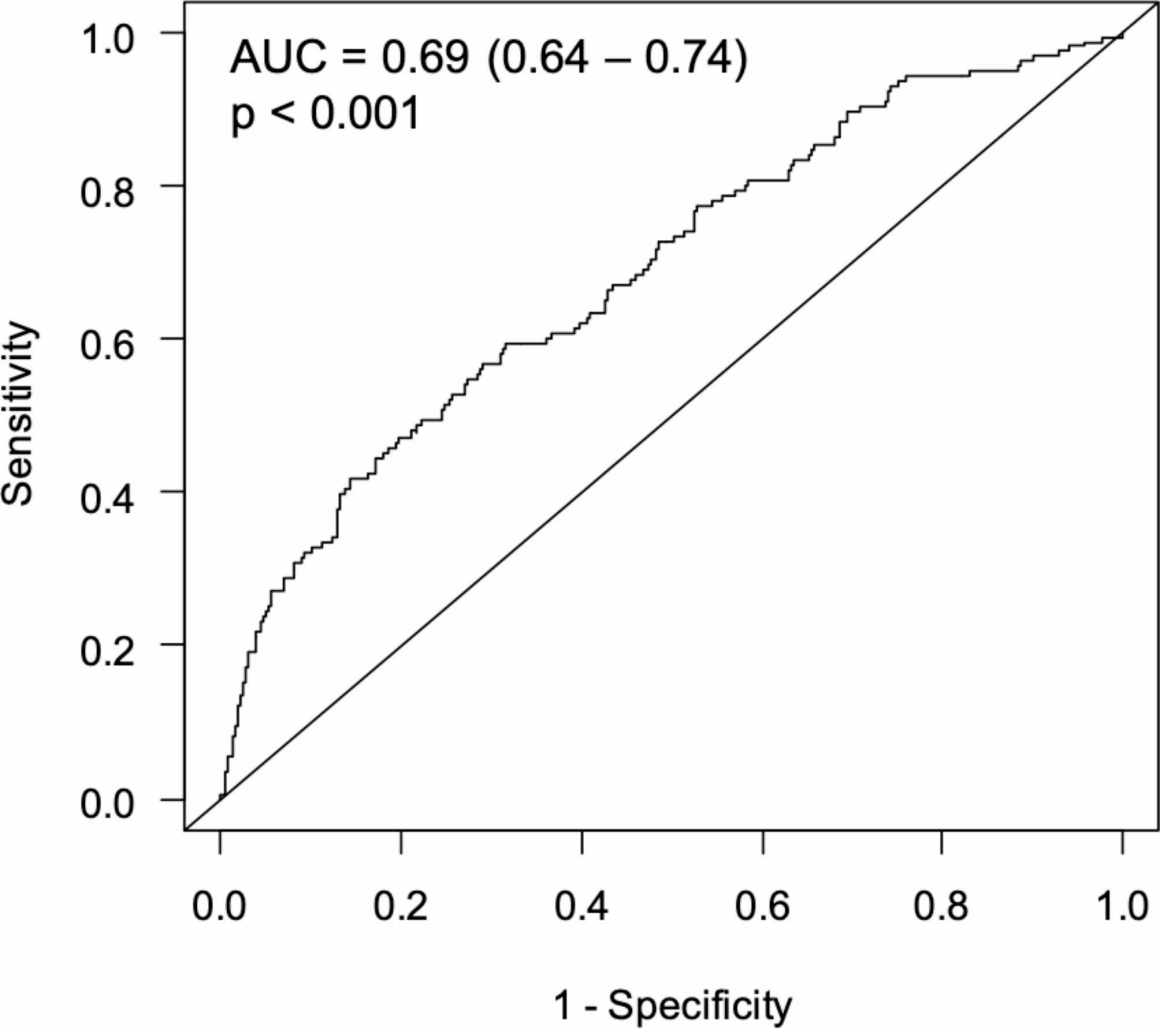
Time-dependent ROC curve for the prediction of total mortality after 13 years by Deceleration Capacity. ROC, receiver operating characteristic; AUC, area under the ROC curve.

Figure 2 shows the time-dependent ROC curve for the prediction of total mortality after 13 years by Deceleration Capacity. With an area under the ROC curve of 0.69 (0.64–0.74, *p* < 0.001), Deceleration Capacity was confirmed as a strong risk predictor in the KORA KMC cohort.

This result was underlined by Cox proportional hazards models with categorized variables (Table 2). In the unadjusted model, DC_category 1_ was associated with a 1.74-fold hazard of all-cause death (95%-CI 1.18–2.60; *p* = 0.005) and DC_category 2_ was associated with a 4.61-fold hazard of all-cause death (95%-CI 3.18–6.70; *p* < 0.001) compared to DC_category 0_. In multivariable model 1 including age, mean heart rate and conventional external risk factors as covariates, DC_category 2_ remained a strong and independent risk predictor with a 2.34-fold hazard of all-cause mortality (95%-CI 1.56–3.51; *p* < 0.001) compared to DC_category 0_ in the investigated cohort. The addition of cardiometabolic conditions, i.e., diabetes mellitus and a history of myocardial infarction, in multivariable model 2 did not alter the hazard ratio for DC_category 2_, showing no evidence of confounding. Cox proportional hazards models using continuous variables where appropriate showed consistent results (Supplementary Table S1). Analyses incorporating interaction terms revealed that there was no significant interaction in predicting mortality between Deceleration Capacity and cardiometabolic conditions (i.e. diabetes mellitus and previous myocardial infarction), age, heart rate, alcohol consumption, and smoking status (Supplementary Tables S2 – S6).

**Fig. 3:**
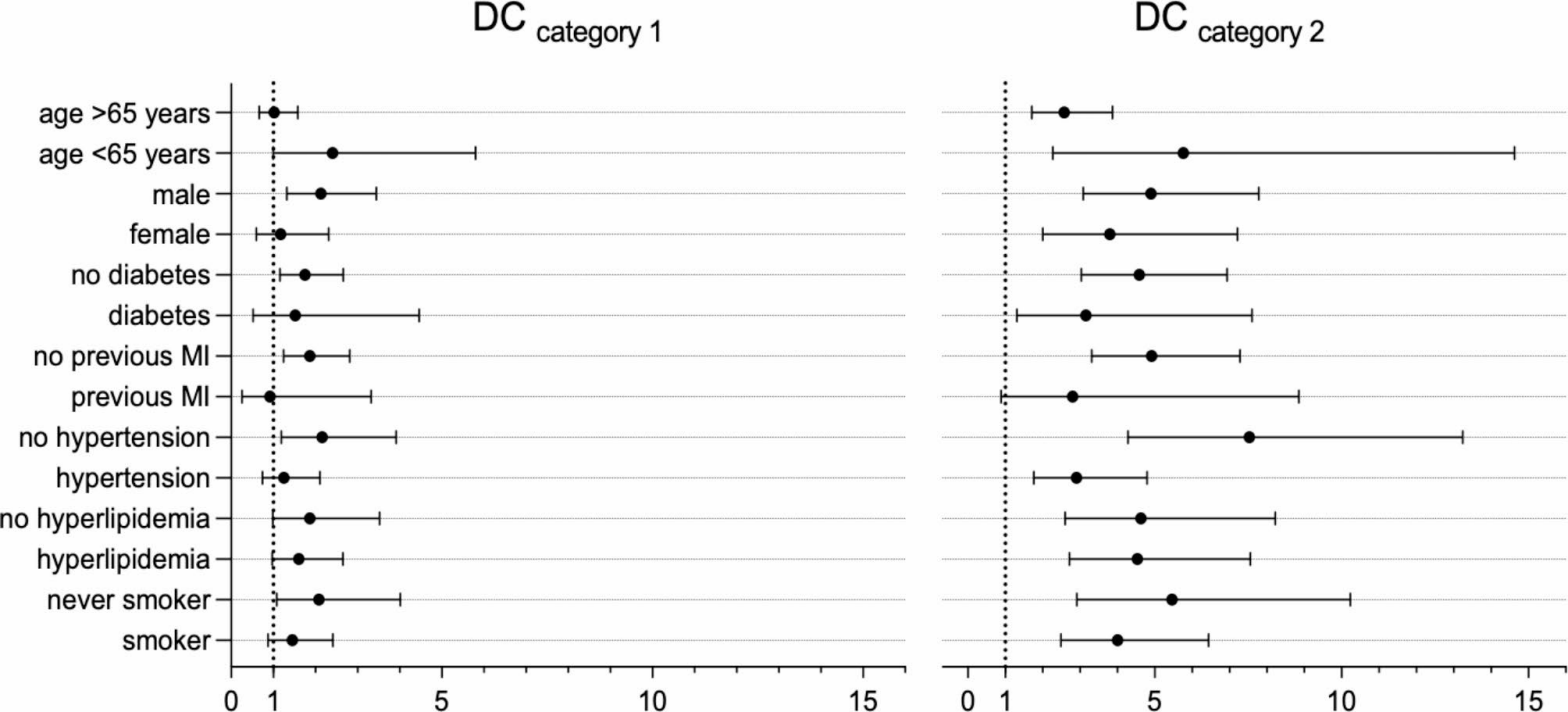
Subgroup analysis showing hazard ratios together with their 95% confidence intervals for the prediction of mortality. The figure shows the result of an univariable Cox regression model comparing both DC_category 1_ and DC_category 2_ with DC_category 0_. MI, myocardial infarction; DC, Deceleration Capacity.

To further investigate the prognostic power of Deceleration Capacity in different population subgroups, the KORA KMC cohort was divided according to age (≥ 65 years), sex (female / male), presence of diabetes mellitus, previous myocardial infarction, presence of arterial hypertension, presence of hyperlipidemia, and smoking status (current or former smokers / never smokers) and additional Cox proportional hazards models were performed. Mortality risk prediction based on Deceleration Capacity was effective in all subgroups analyzed (Fig. 3).

## Discussion

The results of this study confirm that Deceleration Capacity

- can automatically be derived from 5-minute electrocardiogram recordings and
- is an independent and strong predictor of long-term mortality in the general population.

Phase-rectified signal averaging (PRSA)-based Deceleration Capacity was developed and validated with 24 h Holter-electrocardiograms from myocardial infarction survivors^1^. It was introduced as a non-invasive risk stratification tool in these patients that identifies three different mortality risk groups (low, intermediate, and high risk) enabling a more individualized follow-up care focusing on the high-risk patients with the aim of improving outcomes and reducing the socioeconomic disease burden.

So far, Deceleration Capacity has not yet been validated as a non-invasive screening tool in the general population. Such comprehensively applied medical screening tools must meet some requirements^14^: they must be widely available and implementable in the daily clinical routine and therefore be non-invasive and harmless, easy to use, time-effective, and cost-effective. Deceleration Capacity in its original form is obtained from non-invasive 24-hours Holter recordings limiting its scalability and its potential integrability in the daily clinical routine of preventive physicians and general practitioners. To eliminate this limitation within the KORA-KMC cohort, Deceleration Capacity was calculated from scalable short-term electrocardiograms with a duration of only 5-minutes at any time during daytime. Consequently, ultra-low frequency heart rate variability modulations and circadian fluctuations of the autonomic tone were ignored by the measurements within the KORA-KMC study. In addition, the original Deceleration Capacity assessment required experienced technicians or physicians to verify the raw electrocardiogram signals and to eliminate artefacts and correct QRS annotations where needed. By using a fully automated signal processing algorithm, we were capable of improving time- and cost-effectiveness compared to the original approach. Thus, in the here presented form, Deceleration Capacity fulfils some important technical requirements for a general comprehensive medical screening tool.

As a representative population-based cohort study with a very long median follow-up period of 13.4 years and a low drop-out rate, the KORA-KMC study is perfectly suited to validate Deceleration Capacity as a prognostic parameter in the general population. Previously defined cut-off values for the definition of three different risk groups were applied on the KORA-KMC cohort without any further optimization. The values of Deceleration Capacity in the KORA-KMC cohort were within the expected range compared to previous studies^1,2^. Similar to previous studies conducted on myocardial infarction survivors, Deceleration Capacity can differentiate between three risk groups in the general population. It identifies a majority of individuals (more than two-thirds of the study population) with a low mortality risk of 16.7%, compared to a small subgroup (about 10% of the study population) with a significantly higher mortality risk of almost 50% over a follow-up period of more than 13 years. Uni- and multivariable Cox proportional hazards models confirmed Deceleration Capacity as a strong predictor of mortality in the general population that is independent from conventional external risk factors and from cardiometabolic conditions such as diabetes mellitus and a history of myocardial infarction. Importantly, mortality prediction by Deceleration Capacity was successful in all investigated subgroups including subgroups with lower risk or free of underlying cardiometabolic diseases.

Particular attention should be paid to the very high mortality rate during the first years following the determination of Deceleration Capacity (Fig. 1B). The univariable hazard ratio for all-cause death was 11.00 (95%-CI: 4.00–30.27; *p* < 0.001) in DC_category 2_ participants after the first 24 months of follow-up. This suggests that careful and frequent medical supervision of high-risk patients, particularly during the initial years following Deceleration Capacity assessment, may be crucial in significantly improving the prognosis of this group. However, according to the landmark analysis, Deceleration Capacity based mortality risk stratification remains effective even 10 and more years after the initial risk assessment (Supplementary Fig. S1). Implementation of this method as part of a widespread screening program for the general population could facilitate a more targeted and efficient allocation of medical resources within prevention programs, thereby contributing to an overall enhancement of medical care.

Evolving health awareness and the emerging capabilities of wearable medical devices including the assessment of biosignal-derived risk predictors open up entirely new possibilities for risk stratification and screening programs in the general population. Future validation in independent cohorts and prognostic intervention studies will be needed to support the conclusions of this study and to assess the public health benefit of such a preventive approach.

Some limitations of the here presented study have to be considered. First, the KORA-KMC cohort included patients aged 27 to 76 years and therefore, the results are not applicable to older individuals. Secondly, 24-hour Holter electrocardiograms were not obtained in this group of participants meaning that the Deceleration Capacity values cannot be compared with conventional Holter derived values. Thirdly, no follow-up electrocardiograms were available leaving a lack of evidence about the prognostic relevance of serial measurements. Fourthly, although all-cause mortality is a hard endpoint, no conclusion can be drawn regarding specific causes of death.

In conclusion, the fully automated assessment of Deceleration Capacity, derived from a brief 5-minute electrocardiogram recording, emerges as a robust, feasible, and independent predictor of long-term mortality risk in the general population. We advocate for the integration of Deceleration Capacity into comprehensive screening programs for the general population.

## Electronic supplementary material

Supplementary Material 1

## Data Availability

The dataset analysed during the current study is not publicly available due to national data protection laws, since the informed consent given by KORA study participants does not cover data posting in public databases. Data are available upon request by means of a project agreement from KORA. Requests should be sent to kora.passt@helmholtz-munich.de and are subject to approval by the KORA Board.
