# Supplementary material for: Deceleration capacity derived from a five-minute electrocardiogram predicts mortality in the general population
Source: Sci Rep. 2024 Dec 19;14:30566. doi: 10.1038/s41598-024-83712-w (PMC11659320; doi:10.1038/s41598-024-83712-w)
Supplement: Supplementary file 1 — Supplementary Material 1 [file 41598_2024_83712_MOESM1_ESM.pdf]

# Deceleration Capacity Derived from a Five-Minute Electrocardiogram Predicts Mortality in the General Population

Alexander Steger,[1,2,*] Petra Barthel,[1] Alexander Müller,[1] Ina-Maria Rückert-Eheberg,[3] Birgit Linkohr,[2,3] Julia Allescher,[1] Melanie Maier,[1] Alexander Hapfelmeier,[4,5] Eimo Martens,[1] Helene Hildegard Heidegger,[6] Arne Michael Müller,[1] Konstantinos D. Rizas,[2,7] Stefan Kääb,[2,7] Moritz F. Sinner,[2,7] Daniel Sinnecker,[8] Karl-Ludwig Laugwitz,[1,2] Annette Peters[2,3,9,#], Georg Schmidt[1,2,#].

Supplementary Figure S1 Landmark analysis: Kaplan-Meier estimates of mortality stratified by three Deceleration Capacity risk groups using a landmark time of 10 years

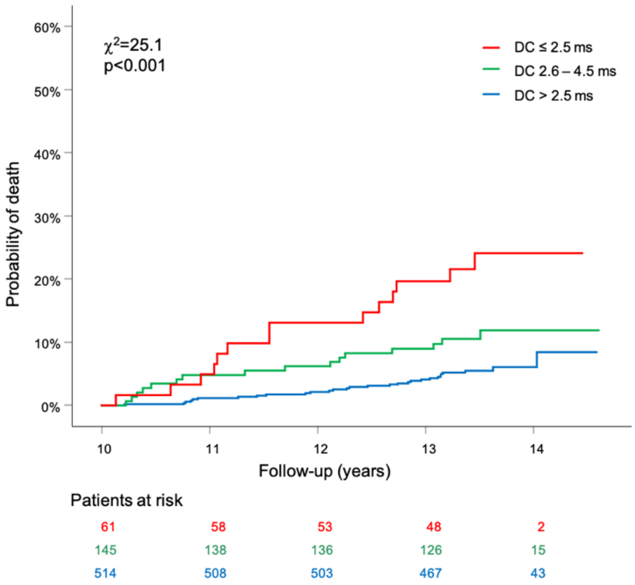


Probabilities of mortality were significantly different between all three Deceleration Capacity risk groups ($p < 0.001$). DC, Deceleration Capacity

Supplementary Table S1 Cox proportional hazards models for the prediction of mortality (continuous variables)

| Variable | Univariable models | | Multivariable model 1 | | Multivariable model 2 | |
|---|---|---|---|---|---|---|
| | HR (95%-CI) | p | HR (95%-CI) | p | HR (95%-CI) | p |
| Age, years | 1.13 (1.11 – 1.16) | <0.001 | 1.14 (1.11 – 1.18) | <0.001 | 1.14 (1.11 – 1.17) | <0.001 |
| Diabetes mellitus | 2.35 (1.54 – 3.61) | <0.001 | *not part of the model* | | 1.44 (0.92 – 2.26) | 0.107 |
| Previous AMI | 2.98 (1.83 – 4.88) | <0.001 | *not part of the model* | | 2.03 (1.22 – 3.39) | 0.007 |
| Mean heart rate, $min^{-1}$ | 1.01 (0.99 – 1.03) | 0.095 | 1.01 (0.99 – 1.03) | 0.086 | 1.02 (1.00 – 1.03) | 0.042 |
| Body mass index, $kg/m^2$ | 1.03 (0.99 – 1.07) | 0.077 | 1.01 (0.98 – 1.05) | 0.465 | 1.00 (0.96 – 1.04) | 0.878 |
| Active smoker | 1.56 (1.00 – 2.44) | 0.049 | 2.78 (1.75 – 4.41) | <0.001 | 2.72 (1.71 – 4.32) | <0.001 |
| Former smoker | 1.36 (0.96 – 1.93) | 0.087 | 1.28 (0.90 – 1.84) | 0.174 | 1.25 (0.87 – 1.79) | 0.232 |
| Alcohol consumption, g/day | 1.01 (1.00 – 1.01) | 0.012 | 1.01 (1.00 – 1.02) | 0.001 | 1.01 (1.00 – 1.02) | 0.003 |
| Deceleration Capacity, ms | 0.90 (0.87 – 0.92) | <0.001 | 0.94 (0.90 – 0.98) | 0.001 | 0.94 (0.90 – 0.97) | 0.001 |

HR, hazard ratio; 95%-CI, 95% confidence interval; AMI, acute myocardial infarction; $min^{-1}$, beats per minute; DC, Deceleration Capacity.

Supplementary Table S2 Multivariable Cox proportional hazards model including interaction terms for Deceleration Capacity and cardiometabolic conditions

| Variable | HR (95%-CI) | p |
|---|---|---|
| Age ≥ 65 years | 6.12 (3.95 – 9.47) | <0.001 |
| Diabetes mellitus | 1.18 (0.59 – 2.34) | 0.645 |
| Previous AMI | 2.71 (1.41 – 5.21) | 0.003 |
| Median Heart Rate ≥ 75 $min^{-1}$ | 1.41 (0.96 – 2.07) | 0.077 |
| Body mass index, $kg/m^2$ | 1.00 (0.96 – 1.04) | 0.924 |
| Active smoker | 2.26 (1.42 – 3.61) | <0.001 |
| Former smoker | 1.25 (0.87 – 1.80) | 0.230 |
| Alcohol consumption, 20 g/day | 1.25 (1.08 – 1.40) | 0.002 |
| $DC_{category\ 1}$ | 1.15 (0.74 – 1.80) | 0.543 |
| $DC_{category\ 2}$ | 2.55 (1.61 – 4.03) | <0.001 |
| $DC_{category\ 1}$ * diabetes mellitus | 1.38 (0.42 – 4.49) | 0.597 |
| $DC_{category\ 2}$ * diabetes mellitus | 0.93 (0.34 – 2.53) | 0.882 |
| $DC_{category\ 1}$ * previous AMI | 0.67 (0.17 – 2.63) | 0.562 |
| $DC_{category\ 2}$ * previous AMI | 0.59 (0.17 – 2.09) | 0.412 |

HR, hazard ratio; 95%-CI, 95% confidence interval; AMI, acute myocardial infarction; $min^{-1}$, beats per minute; DC, Deceleration Capacity.

Supplementary Table S3 Multivariable Cox proportional hazards model including interaction terms for Deceleration Capacity and age

| Variable | HR (95%-CI) | p |
|---|---|---|
| Age ≥ 65 years | 8.04 (4.61 – 14.0) | <0.001 |
| Diabetes mellitus | 1.18 (0.75 – 1.87) | 0.469 |
| Previous AMI | 2.20 (1.32 – 3.65) | 0.002 |
| Mean heart rate ≥ 75 $min^{-1}$ | 1.38 (0.94 – 2.03) | 0.098 |
| Body mass index, $kg/m^2$ | 0.99 (0.96 – 1.04) | 0.976 |
| Active smoker | 2.26 (1.42 – 3.59) | <0.001 |
| Former smoker | 1.22 (0.85 – 1.76) | 0.280 |
| Alcohol consumption, 20 g/day | 1.23 (1.08 – 1.40) | 0.002 |
| $DC_{category\ 1}$ | 2.01 (0.82 – 4.93) | 0.127 |
| $DC_{category\ 2}$ | 4.11 (1.58 – 10.7) | 0.004 |
| $DC_{category\ 1}$ * age ≥ 65 years | 0.50 (0.18 – 1.35) | 0.169 |
| $DC_{category\ 2}$ * age ≥ 65 years | 0.52 (0.19 – 1.43) | 0.204 |

HR, hazard ratio; 95%-CI, 95% confidence interval; AMI, acute myocardial infarction; $min^{-1}$, beats per minute; DC, Deceleration Capacity.

Supplementary Table S4 Multivariable Cox proportional hazards model including interaction terms for Deceleration Capacity and heart rate

| **Variable** | **HR (95%-CI)** | **p** |
|---|---|---|
| Age ≥ 65 years | 6.16 (3.98 – 9.51) | <0.001 |
| Diabetes mellitus | 1.17 (0.74 – 1.86) | 0.494 |
| Previous AMI | 2.13 (1.28 – 3.55) | 0.004 |
| Mean heart rate ≥ 75 $min^{-1}$ | 2.05 (1.16 – 3.64) | 0.014 |
| Body mass index, $kg/m^2$ | 1.00 (0.96 – 1.04) | 0.908 |
| Active smoker | 2.24 (1.41 – 3.56) | <0.001 |
| Former smoker | 1.22 (0.85 – 1.75) | 0.294 |
| Alcohol consumption, 20 g/day | 1.24 (1.08 – 1.41) | 0.001 |
| $DC_{category\ 1}$ | 1.33 (0.85 – 2.07) | 0.214 |
| $DC_{category\ 2}$ | 2.63 (1.63 – 4.24) | <0.001 |
| $DC_{category\ 1}$ * mean HR ≥ 75 $min^{-1}$ | 0.48 (0.18 – 1.26) | 0.133 |
| $DC_{category\ 2}$ * mean HR ≥ 75 $min^{-1}$ | 0.62 (0.27 – 1.41) | 0.255 |

HR, hazard ratio; 95%-CI, 95% confidence interval; AMI, acute myocardial infarction; $min^{-1}$, beats per minute; DC, Deceleration Capacity; HR, heart rate.

Supplementary Table S5 Multivariable Cox proportional hazards model including interaction terms for Deceleration Capacity and alcohol consumption

| Variable | HR (95%-CI) | p |
|---|---|---|
| Age ≥ 65 years | 6.34 (4.09 – 9.82) | <0.001 |
| Diabetes mellitus | 1.17 (0.74 – 1.84) | 0.509 |
| Previous AMI | 2.20 (1.33 – 3.65) | 0.002 |
| Mean heart rate ≥ 75 $min^{-1}$ | 1.42 (0.97 – 2.08) | 0.070 |
| Body mass index, $kg/m^2$ | 1.00 (0.96 – 1.04) | 0.942 |
| Active smoker | 2.25 (1.41 – 3.58) | <0.001 |
| Former smoker | 1.25 (0.87 – 1.80) | 0.223 |
| Alcohol consumption, 20 g/day | 1.24 (1.03 – 1.50) | 0.026 |
| $DC_{category\ 1}$ | 0.96 (0.58 – 1.59) | 0.878 |
| $DC_{category\ 2}$ | 2.77 (1.69 – 4.52) | <0.001 |
| $DC_{category\ 1}$ * alcohol $consumption_{20\ g/d}$ | 1.19 (0.89 – 1.58) | 0.249 |
| $DC_{category\ 2}$ * alcohol $consumption_{20\ g/d}$ | 0.85 (0.62 – 1.16) | 0.299 |

HR, hazard ratio; 95%-CI, 95% confidence interval; AMI, acute myocardial infarction; $min^{-1}$, beats per minute; DC, Deceleration Capacity.

Supplementary Table S6 Multivariable Cox proportional hazards model including interaction terms for Deceleration Capacity and smoking status

| Variable | HR (95%-CI) | p |
|---|---|---|
| Age ≥ 65 years | 6.11 (3.95 – 9.45) | <0.001 |
| Diabetes mellitus | 1.17 (0.74 – 1.85) | 0.512 |
| Previous AMI | 2.25 (1.34 – 3.76) | 0.002 |
| Mean heart rate ≥ 75 $min^{-1}$ | 1.43 (0.98 – 2.10) | 0.067 |
| Body mass index, $kg/m^2$ | 1.00 (0.97 – 1.04) | 0.824 |
| Active smoker | 2.45 (1.23 – 4.86) | 0.010 |
| Former smoker | 1.54 (0.91 – 2.51) | 0.108 |
| Alcohol consumption, 20 g/day | 1.23 (1.08 – 1.40) | 0.002 |
| $DC_{category\ 1}$ | 1.27 (0.66 – 2.46) | 0.476 |
| $DC_{category\ 2}$ | 3.20 (1.66 – 6.15) | <0.001 |
| $DC_{category\ 1}$ * active smoker | 1.31 (0.46 – 3.76) | 0.614 |
| $DC_{category\ 2}$ * active smoker | 0.57 (0.19 – 1.75) | 0.326 |
| $DC_{category\ 1}$ * former smoker | 0.65 (0.26 – 1.65) | 0.366 |
| $DC_{category\ 2}$ * former smoker | 0.65 (0.28 – 1.50) | 0.307 |

HR, hazard ratio; 95%-CI, 95% confidence interval; AMI, acute myocardial infarction; $min^{-1}$, beats per minute; DC, Deceleration Capacity.
